# Challenges and Solutions for Functional Neurosurgery in Developing Countries

**DOI:** 10.7759/cureus.3314

**Published:** 2018-09-17

**Authors:** Francis Fezeu, Arjun Ramesh, Patrick D. Melmer, Shayan Moosa, Paul S Larson, Fraser Henderson

**Affiliations:** 1 Neurology, BRAIN Global, Charlottesville, USA; 2 Anesthesiology, Rush University Medical Center, Chicago, USA; 3 Surgery, Grand Strand Medical Center, Myrtle Beach, USA; 4 Neurosurgery, University of Virginia Health System, Charlottesville, USA; 5 Neurological Surgery, University of California, San Francisco, USA; 6 Neurosurgery, Medical University of South Carolina, Charleston, USA

**Keywords:** epilepsy surgery, functional neurosurgery, global surgery, low and middle income countries, global neurosurgery

## Abstract

Functional neurosurgery techniques remain integral to the neurosurgical treatment armamentarium but data on global implementation remains scarce. In comparison to high-income countries (HIC), low- and middle-income countries (LMIC) suffer from an increased prevalence of diseases like epilepsy, which may be amenable to functional techniques, and therefore, LMIC may benefit from an increased utilization of these treatment modalities. However, functional techniques tend to be expensive and thus difficult to implement in the LMIC setting. A review was performed to assess the current status of functional neurosurgical techniques in LMIC as a starting point for future initiatives.

For methodology, a review of the current body of literature on functional neurosurgery in LMIC was conducted through the United States National Library of Medicine Pubmed search engine. Search terms included “functional neurosurgery,” “developing countries,” “low and middle income,” and other related terms.

It was found that though five billion people lack access to safe surgical care, the burden of disease amenable to treatment with functional neurosurgical procedures remains unknown. Increasingly, reports of successful, long-term, international neurosurgical collaborations are being reported, but reports in the sub-field of functional neurosurgery are lacking.

In conclusion, awareness of global surgical disparities has increased dramatically while global guidelines for functional techniques are currently lacking. A concerted effort can harness these techniques for wider practice. Partnerships between centers in LMIC and HIC are making progress to better understand the burden of disease in LMIC and to create context-specific solutions for practice in the LMIC setting, but more collaborations are warranted.

## Introduction and background

For most of the 20th century and increasingly so in the 21st century, functional procedures have remained integral to the neurosurgical armamentarium, allowing the treatment of many pathologies, including epilepsy, Parkinson’s disease, other movement disorders, pain syndromes, and some psychiatric disorders. However functional surgeries may require particularly extensive and expensive resources, creating a barrier to the adoption of functional techniques for low- and middle-income countries (LMIC) that are home to the majority of the five-billion people lacking access to safe surgical care [1]. Admittedly, stroke and traumatic brain injury dwarf other neurosurgical conditions with respect to projected impact on the gross domestic product [2]. Nevertheless, increasingly, the worldwide need for surgical services has been recognized, even as statistics for worldwide neurosurgical needs have been slower to emerge [3]. Not simply functional neurosurgery but all neurosurgical subspecialties, including skull base techniques for brain tumors or cerebrovascular endovascular techniques for acute stroke treatment, face similar global disparity in implementation [4]. Recently, the Global Spine Care Initiative has published an extensive outline based upon eight principles that may direct a new generation of spinal surgery in LMIC [5]. As in spine surgery, the burden of disease is higher in LMIC for conditions amenable to functional procedures, for example, the prevalence of epilepsy is higher in LMIC than in high-income countries (HIC) [6]. It is, therefore, imperative to creatively harness functional neurosurgical techniques for common use in LMIC.

LMIC lack trained neurosurgeons. The ratio of neurosurgeons to individuals in the majority of HIC is one to two hundred thousand; neurosurgeons in LMIC, with a ratio of one to six million four hundred thousand patients, may be responsible for a population 30 times greater than their HIC counterparts [7-8]. A recent worldwide analysis determined that five-million individuals suffering from treatable “essential neurosurgical cases” will never undergo the indicated procedure, also finding that most of these cases will occur in Africa or Southeast Asia [4]. It is easy to extrapolate that surgeons with sub-specialty training in functional and stereotactic “non-essential” techniques, such as for movement disorders, are in even greater demand. Advanced technologies that are readily found in HIC are also lacking in LMIC, making performing functional neurosurgery difficult in these regions. Therefore, in order to bring about the most effective treatment of myriad neurological diseases that can effectively be treated by functional neurosurgery, the standardization of techniques within specific resource contexts may facilitate wider global implementation.

There is a paucity of literature on functional neurosurgery in LMIC, though the problem is now being framed with increasingly sophisticated digital surveys [4]. However, these regions stand to gain considerably from further studies. Given the burden of disease in these regions, further action by the global community is required. A recent survey-based study of expert opinion acknowledged that 40% of epilepsy cases may warrant neurosurgical consultation and 24% of cases may warrant intervention [9]. In this report, we review literature related to the current status of functional neurosurgery practices in LMIC to justify the necessity for immediate action in this field.

## Review

The quantification of specific disease burdens in LMIC regions, while historically difficult, is made easier using new technology [10]. It is well known that these regions suffer from a higher burden of epilepsy, to take one example, than HIC [6]. Additionally, many patients with a surgically correctable disease may not present to a neurosurgeon for a long time due to cultural differences and a shortage of neurosurgeons [11]. For patients with tumors that invade eloquent regions of the brain, current practices in LMIC either result in high rates of morbidity or a decision not to operate, whereas capabilities for awake craniotomy facilitate better outcomes in surgery on eloquent regions of the brain [12].

Standardization of procedures tailored to LMIC

Functional procedures have historically lacked standard protocols though national societies have begun publishing these [13-14]. Often, these procedures are conducted based on institutional tradition and surgeon preference. In other fields, guidelines have been shown to improve outcomes by standardizing care [15], and there is no reason to suspect this would be different in functional neurosurgery, provided that protocols are tailored to a specific hospital's regional context. While one protocol may not be standardizable across the globe, there may be comparable tiers across which protocols may be shared. A recent study reviewing esophagectomy outcomes in a "safety net" hospital treating a sicker cohort of patients in the United States demonstrated decreased complications in comparison to national averages that was attributed to the institution of a protocol [16].

There have been a few studies that demonstrate this lack of standardization. One study, which surveyed epilepsy centers worldwide, found that there was a wide range in the practice of mapping techniques. The survey found large variations, ranging from different electrical stimulation settings to the types of language function tested in the patient, and from their institution’s definition of a “defect in language” to the size of their surgical resections [13]. Another study surveying the literature found that there was no consensus on the best method of anesthesia in awake craniotomies, with a lack of standardized protocols. Furthermore, due to the lack of standardization, no meaningful comparisons could be made to better understand the best methods for inducing anesthesia during awake craniotomies [17].

The Pediatric Epilepsy Surgery Commission of the Commission on Neurosurgery of the International League Against Epilepsy conducted a global survey in order to create guidelines for epilepsy surgery in children [18]. In this survey, 20 programs from the United States, Europe, and Australia were asked details of their standards for the care of pediatric patients undergoing functional neurosurgery for epilepsy refractory to non-surgical treatment. In nearly half of the patients treated by these centers (46%), the age of the child’s first seizure was 12 months old or younger, and just over a quarter of the patients (27%) received intracranial electrodes during their surgery. The most common type of surgery undergone by these pediatric patients was a lobar and focal resection of the frontal and temporal lobes (41%), followed then by cerebral hemispherectomy and vagal nerve stimulation (16% each) and multilobar resections (13%). The centers within the United States conducted a greater number of vagal stimulation surgeries than those in Europe or Australia, and they also operated more often going off of fewer magnetic resonance imaging (MRI) findings in the patients but utilized intracranial electrodes and neuroimaging more frequently than the other two regions [18]. These data present a compelling glance at the diverse protocols in HIC and could be useful in the development of worldwide standards to be utilized by LMIC doing pediatric functional neurosurgery.

The implementation of guidelines could help to provide the framework for the large-scale implementation of functional techniques in LMIC. With guidelines in place, centers can begin to procure the equipment required, knowing that all neurosurgeons will use similar techniques. Taking the example of awake craniotomies, it has been suggested that the economic benefits of conducting these procedures may help the economies of LMIC [19]. Additionally, local context-specific innovation from the framework of the global guidelines can help guide further resource-sparing interventions that can be expanded globally. This innovation can help modify management in both the HIC and LMIC contexts to provide improved outcomes and cost-effective treatments to patients worldwide [15].

Teaching functional neurosurgery procedures in LMIC

It is feasible to teach standards of practice to neurosurgeons in LMIC, and the potential for increased outcomes is great. Bernstein, with experience from more than 1,000 awake craniotomies, trained neurosurgeons from China, Indonesia, Ghana, and Nigeria, operating on a total of 38 using awake craniotomy techniques [19]. No deaths occurred among these surgical patients and 64% of the patients were in the hospital for fewer than 10 days. Furthermore, no cases required urgent intubation and there was only a single case of permanent postoperative deficit. This experience demonstrates that standardized protocols can be successfully implemented in LMIC and that functional neurosurgery is feasible with the equipment already available in some countries.

However, standardization of technique is not enough. There must also be a collaboration with centers in HIC to bring functional techniques to LMIC. Tunisia, the northernmost country within the African continent, established its first epilepsy center in the late 2000s following collaboration with the departments of neurosurgery and neurophysiology at Charles Nicolle Hospital in Rouen, France [20]. Fifteen patients with mesial temporal lobe epilepsy, refractory to oral medications, were included in the original study, 10 of whom underwent surgery. Patients were noted to be seizure-free in 100% of the cases, with no operative mortality or major complications. In another study, several researchers sought to characterize the success of an epilepsy program in the resource-poor setting of Uganda in Africa, specifically at the CURE Children’s Hospital of Uganda [6]. Forty-nine patients were evaluated from 2005 to 2007, with a mean age of 13 years. Ten of these patients met criteria to undergo corticoamygdalohippocampectomy. Sixty percent of the patients were seizure-free following the surgery, 20% experienced a reduction in seizure frequency of greater than 90%, and the final 20% experienced a similar frequency of seizure but of a less severe nature. At the King Faisal Hospital in Rwanda, the first awake neurosurgery was performed successfully in collaboration with neurosurgeons from Duke in 2011 [21]. Recently, through a collaboration with United Kingdom-based practitioners, a spinal cord injury rehabilitation center has been established in Madagascar [22]. Here lie several excellent examples of successful collaboration between an established neurosurgical center and one being developed in an LMIC within the African continent. Emulation of this process is indeed feasible around the globe.

Recommendations

It is difficult, in the developing world, to quantify the need for neurological surgery in its varying degrees of criticality, let alone that of functional neurosurgery [2,4,23]. However, there is undoubtedly an unmet need in these regions, making it imperative to bring functional techniques to LMIC. Current barriers to care include a scarcity of surgeons trained in functional techniques and a lack of equipment needed to perform them. Increased education and access are certainly required in these regions.

Importantly, standardized practice guidelines tailored to lower-resource settings will be needed for functional techniques. Guidelines are relatively new in the HIC context, and perhaps these may be modified and deployed in LMIC [14]. To this end, prospective trials, investigating different techniques, are needed in HIC. Although such trials are already underway at some centers, a large accepted set of guidelines from organizations such as the American Society for Stereotactic and Functional Neurosurgery (ASSFN) or the World Society for Stereotactic and Functional Neurosurgery (WSSFN) will be needed.

Collaborations between HIC and LMIC are needed to help improve the state of functional neurosurgery in resource-limited regions. These collaborations can help bring functional techniques to LMIC through physician exchanges and mentorship. There are already several collaborations, which have helped to bring functional techniques to the developing world. However, further collaborations are still needed. Additionally, research focusing on the unique disease profiles in these regions is needed to help demonstrate the current need and secure further funding for research and innovation. 

Improvements are also needed to augment the technological capacity in these regions. Currently, it is difficult for LMIC to procure the expensive equipment required for functional neurosurgery [24-25]. Donations from HIC help this problem to an extent, but there are limitations in repairing equipment in LMIC, with months passing before a qualified technician can come to fix the problems. With increased research demonstrating to the device companies that there will be a strong utilization of their equipment in these regions, the device companies may invest in stationing technicians in these regions, in turn, allowing for improved technology in surgery.

Ultimately, in the LMIC context, resource allocation will be an important issue to resolve [26]. A better understanding of disease burden will be needed in order to help direct resources to where they are needed to most. Collaborations with research centers in HIC will be crucial to address this issue. Additionally, an increased supply of neurosurgeons will be needed to allow for increased specialization without compromising patient care. 

Implementing functional techniques in LMIC will be of benefit in both the LMIC and HIC contexts. Context-specific innovation in LMIC may generate new, resource-sparing techniques, which can be applied in the HIC context. With the current trend of rising health care costs, innovation to reduce the overall cost of interventions is imperative, regardless of practice setting. Innovation from LMIC has resulted in an improvement in other fields of neurosurgery and there is no reason to suspect functional neurosurgery will be any different.

## Conclusions

As with many technological advances, those in functional neurosurgery lag in implementation to LMIC. Diseases that may be effectively treated through the use of functional and stereotactic neurosurgery include epilepsy, Parkinson’s disease, and even some psychiatric disorders, among others. The need for neurosurgeons is great throughout the world, however, and the vast majority of the world’s neurosurgeons are unable to care for a large portion of the world’s population. This discrepancy is most evident in LMIC, where access to care is limited by geography, training, economic resources, practice disparity, and absent infrastructure. We have examined several examples of hope for this seemingly bleak situation, however, and there have been successes in the tailored standardization of neurosurgical procedures and education of neurosurgeons in various LMIC to bring about positive change. A comprehensive, collaborative, international strategy remains to be identified.
